# Childhood

**Published:** 1894-03

**Authors:** 


Childhood: The Magazine for Parents. Edited by George Wm. Winterburn, M. D., and Florence Hull. New York: Childhood Publishing Co., 78 Maiden Lane. Price, $1.00 per year; 10 cents per number.

The January number has been received and is an especially full and most satisfactory issue of this excellent publication. In looking over the table of contents the first article one would think is the most interesting; but on continuing the review this can hardly be said, for they all are equally pleasing and instructive, and especially useful and interesting to parents. Those who do not have the magazine should subscribe, and they will experience the pleasure that its appearance will produce when the month comes around and it appears in their mail matter.

